# Vacuolization in Myeloid and Erythroid Precursors in a Child with Menkes Disease

**DOI:** 10.4274/tjh.galenos.2018.2018.0104

**Published:** 2019-08-02

**Authors:** Seçil Sayın, Şule Ünal, Mualla Çetin, Fatma Gümrük

**Affiliations:** 1 Hacettepe University Faculty of Medicine, Department of Child Health and Diseases, Unit of Hematology, Ankara, Turkey

**Keywords:** Menkes disease, Copper deficiency, Vacuolization, Bone marrow

A 5-year-old boy who was in follow-up with a clinical and biochemical diagnosis of Menkes disease (MD) since 10 months of age was admitted with diarrhea. On examination he had a characteristic cherubic face, hypopigmented and sparse hair, hepatosplenomegaly, and hypotonia with brisk deep tendon reflexes. A complete blood count revealed the following: hemoglobin, 5.5 g/dL; hematocrit, 16.2%; red blood cells, 1.69x10^12^/L; mean corpuscular volume, 95.8 fL; mean corpuscular hemoglobin, 32.3 pg; red blood cell distribution width, 19.2%; white blood cells, 2.2x10^9^/L; and platelet count, 157x10^9^/L. Serum vitamin B12 level was 575 pg/mL. Serum copper level was 81 µg/dL and serum zinc level was 152 µg/dL. Peripheral blood smear revealed 34% polymorphonuclear leukocytes, 62% lymphocytes, and 4% monocytes. Bone marrow examination revealed normocellular marrow with megaloblastic changes and widespread cytoplasmic vacuolization in myeloid and erythroid progenitors (Figure 1).

Menkes disease is a neurodegenerative disorder due to mutations in the *ATP7A* gene, which ends with deficiency of copper-dependent enzymes [1].

Cytoplasmic vacuoles of myeloid and erythroid lineages have been described in patients with copper deficiency [2], Pearson syndrome [3], and acute alcoholic intoxication [4]. There have also been reports of megaloblastic changes in copper deficiency [2]. Herein, we exhibited both erythroid and myeloid vacuolizations and severe megaloblastic changes together in a patient with MD. All of these morphological findings in our patient were attributed to copper deficiency.

## Figures and Tables

**Figure 1:**
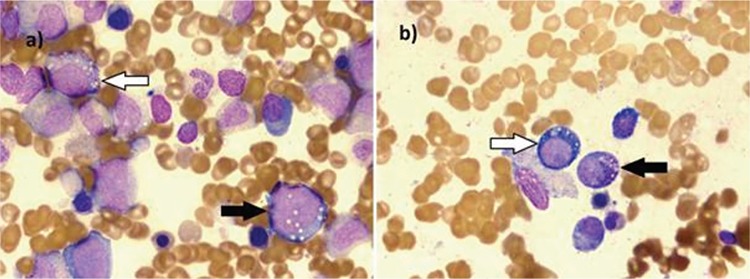
Bone marrow aspiration smears: a) cytoplasmic vacuolization in myeloid precursors (white arrow) and erythroid precursors (black arrow); b) cytoplasmic vacuolization in myeloid precursors (black arrow) and erythroid precursors (white arrow). May-Grünwald Giemsa stain, original magnification 100^x^.
